# Fusaproliferin, a Fungal Mycotoxin, Shows Cytotoxicity against Pancreatic Cancer Cell Lines

**DOI:** 10.3390/molecules23123288

**Published:** 2018-12-11

**Authors:** Nazia Hoque, Choudhury Mahmood Hasan, Md. Sohel Rana, Amrit Varsha, Md. Hossain Sohrab, Khondaker Miraz Rahman

**Affiliations:** 1 Department of Pharmacy, East West University, Dhaka 1212, Bangladesh; nzh@ewubd.edu; 2 Department of Pharmacy, Jahangirnagar University, Savar, Dhaka 1342, Bangladesh; sohelrana.ju@gmail.com; 3 Pharmaceutical Sciences Research Division (PSRD), BCSIR Laboratories, Dhaka 1205, Bangladesh; 4 Department of Pharmaceutical Chemistry, University of Dhaka, Dhaka-1000, Bangladesh; cmhasan@gmail.com; 5 School of Cancer and Pharmaceutical Science, King’s College London, 150 Stamford Street, London SE1 9NH, UK; amritvarsha7dec@gmail.com

**Keywords:** endophytic fungi, sesterterpene, cytotoxic activity, pancreatic cancer

## Abstract

As a part of our ongoing research on endophytic fungi, we have isolated a sesterterpene mycotoxin, fusaproliferin (FUS), from a *Fusarium solani* strain, which is associated with the plant *Aglaonema hookerianum* Schott. FUS showed rapid and sub-micromolar IC_50_ against pancreatic cancer cell lines. Time-dependent survival analysis and microscopy imaging showed rapid morphological changes in cancer cell lines 4 h after incubation with FUS. This provides a new chemical scaffold that can be further developed to obtain more potent synthetic agents against pancreatic cancer.

## 1. Introduction

Pancreatic adenocarcinoma is a leading cause of adult cancer mortality. It is presently untreatable, with a 5-year survival rate of ~5% [1]. As early detection is difficult, most patients present with locally advanced or metastatic disease [2]. Therapeutic options are limited, and metastatic disease frequently develops after surgery [3]. Pancreatic cancer is the seventh leading cause of cancer-related deaths worldwide, and annually more than 200,000 deaths are attributed to pancreatic cancer every year [4,5]. Natural sources, particularly plants, represent an important source of new anticancer chemical scaffolds, and there is an increasing interest in searching for natural products with drug-like properties as potential leads for drug discovery projects [6,7].

Endophytic fungi are symbiotically associated with plants, capable of synthesizing bioactive compounds without causing any damage to the host [8]. Some of these compounds have proven useful for novel drug discoveries, and provide a defense against harmful pathogens for the plants [9,10]. As a part of our ongoing research on endophytic fungi [11,12,13,14], a *Fusarium solani* strain, isolated from the plant *Aglaonema hookerianum* Schott. (Family: Araceae), was investigated. *F. solani* has been proved as a potent source of structurally-diverse natural compounds with cytotoxic activity, such as karuquinone A and karuquinone B [15], 9-desmethylherbarine, 7-desmethylscorpinone and 7-desmethyl-6-methylbostrycoidin [13], camptothecin and 10-hydroxycamptothecin [16], as well as paclitaxel [17]. *F. solani* is considered a plant pathogen, and it accounts for more than 50% infections caused by *Fusarium* spp. Infections by other *Fusarium* spp. strains are relatively uncommon [18] Chemical investigation of the ethyl acetate extract of the *F. solani* led to the isolation of fusaproliferin (FUS), a mycotoxin which was first isolated from the Italian *F. proliferatum* strains, named “proliferin” [19] and later “fusaproliferin” [20]. The absolute stereochemistry of the compound was confirmed by Santini et al. in 1996 [21]. FUS is also produced by *Fusarium subglutinans* and fifteen other ex-type strains of *Fusarium* species [22,23]. FUS produced a toxic effect on *Artemia salina*, insect cells, and human B lymphocytes [24]. It was also reported to produce a teratogenic effect on chicken embryos [25]. In this study, we examined the anticancer activity of FUS against two pancreatic and two breast cancer cell lines, and compared the activity of the compound with that of gemcitabine and doxorubicin, the current drugs of choice for pancreatic and breast cancer, respectively.

## 2. Results and Discussion

**Figure 1:**
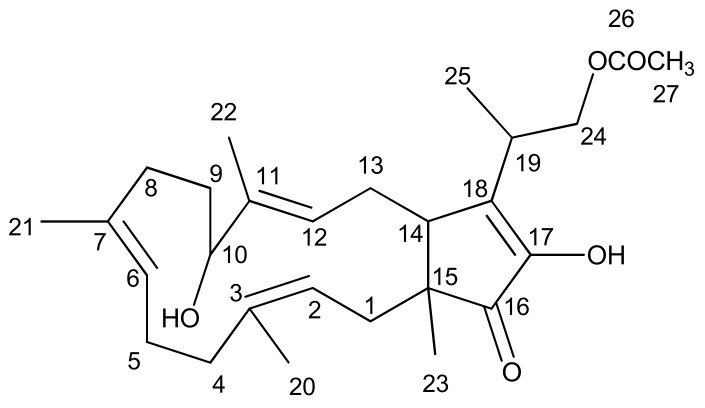
Structure of Fusaproliferin.

FUS was obtained as a white gum. The structure of FUS (Figure 1) was confirmed by spectroscopic analysis (^1^H, ^13^C-NMR, DEPT-135, 2D-NMR and HR-ESIMS) and by comparison with the published spectral values [19]. Accurate mass measurement of FUS obtained by FT-ESI-MS yielded a parent mass at *m*/*z* 467.2778 in positive ionization mode, corresponding to the sodium adduct [M + Na]^+^ with a molecular formula of C_27_H_40_O_5_ (calcd. mass 467.2773, [C_27_H_40_O_5_ + Na]^+^), accounting for 8 degrees of unsaturation. The resonances at *δ* 170.9 and 207.9 ppm in the ^13^C-NMR spectrum were characteristic of the presence of two carbonyl carbons of an ester and a ketone, respectively. The ^1^H and ^13^C-NMR data, in conjunction with the DEPT-135 spectrum (Figure S13), proved the presence of 27 carbon atom signals corresponding to six methyls (20-, 21-, 22-, 23-, 25-, and 27-), seven sp^3^ methylenes (1-, 4-, 5-, 8-, 9-, 13- and 24-), three sp^3^ methines (10-, 14- and 19-), three sp^2^ methines (2-,6- and 12-), one sp^3^ quaternary carbon (15-), five sp^2^ quaternary carbons (3-, 7-, 11-, 17- and 18-), two carbonyl carbons of an ester (26-OCOCH_3_) and a ketone (16-CO-). The presence of three sp^2^ methines and five sp^2^ quaternary carbons, along with one each of an ester and carbonyl moiety, proved the presence of six double bonds in this compound, and thus indicated that it was a bicyclic compound. After deducting the acetyl moiety ‘OCOCH_3_’ (*δ*_H_ = 2.06, *δ*_C_ = 20.9 and 170.9 ppm), the compound consisted of 25 carbons, which indicated it as a sesterterpene.

**Figure 2:**
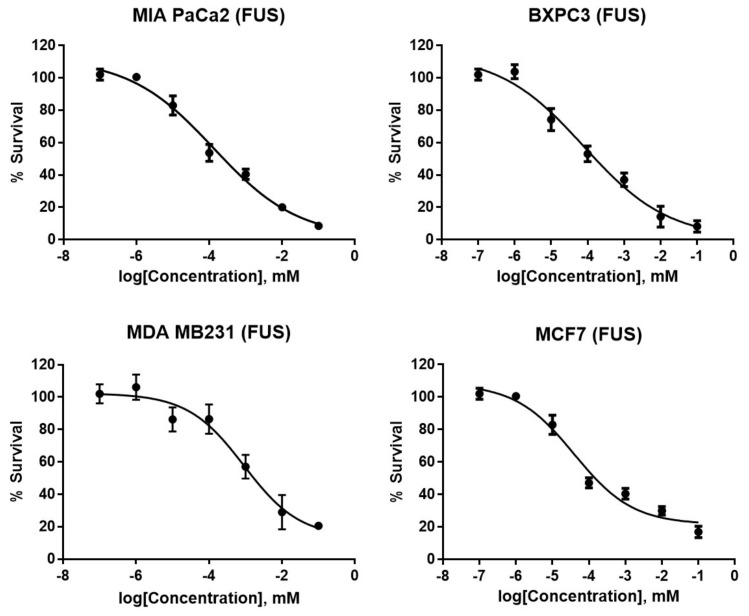
MTT cell-viability assay profile in pancreatic (MIA PaCa2 and BXPC3) and breast (MDA MB 231 and MCF7) cancer cell lines treated with FUS for 24 h.

The cytotoxicity of FUS was determined against two pancreatic cancer cell lines, BxPc3 and MIA PaCa2, the ER-positive breast cancer cell line MCF7, and the triple negative breast cancer cell line MDA MB 231; gemcitabine was used as the positive control for the pancreatic cancer cell lines and doxorubicin for the breast cancer cell lines. FUS was active against all four cell lines tested (Figure 2 and Table 1) with sub to low micromolar IC_50_, but the activity against the pancreatic cancer cell lines were notably better than the breast cancer cell lines. FUS was between 3 to 58 times more potent than gemcitabine in pancreatic cancer cell lines, but doxorubicin was superior against both breast cancer cell lines compared to FUS. The therapeutic utility of FUS was further investigated against WI38, a non-tumor lung fibroblast cell line. FUS was found to be cytotoxic against WI38 with a high micromolar IC_50_ (Table 1), but was between 23 to 138 times more selective for the pancreatic cancer cell lines, and between 4.6 to 9.4 more selective for the breast cancer cell lines. This suggests a good therapeutic index against the pancreatic cancer cell lines which can be exploited if FUS is considered as a starting point for a medicinal chemistry program to develop a more potent analogue. 

**Figure 3:**
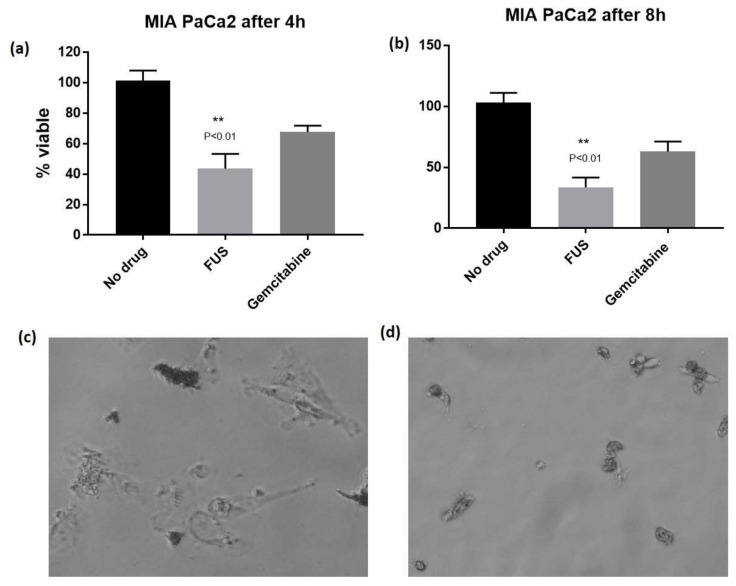
Effect of FUS on pancreatic cancer cell line. (**a**,**b**) FUS showed statistically significant rapid toxicity (*p* < 0.01) against MIA PaCa2 cell line after 4 h and 8 h incubation; (**c**,**d**) morphological changes observed in MIA PaCa2 cell lines after 4 h and 8 h incubation, respectively.

The relatively rapid cytotoxicity observed for FUS during the cell culture experiments led us to carry out a time-dependent cytotoxicity assay by monitoring percentage survival after 4- and 8-h post-incubation. FUS showed greater toxicity at both 4 and 8 h at 4 × IC_50_concentration in MIA PaCa2 cell line compared to gemcitabine. The differences were statistically significant (*p* < 0.01) (Figure 3a). Similarly, rapid toxicity was observed against MDA MB 231 cell line at 4 h (*p* < 0.03) and at 8 h (*p* < 0.01) compared to doxorubicin (Figures S5 and S6), although doxorubicin was notably more potent than FUS after 24 h incubation. 

The morphological changes in the MIAPaCa2 cell line after incubating with FUS were monitored using a Nikon TS100 inverted microscope fitted with a camera. The cells appeared to show both apoptotic and necrotic damages within 4 h post-incubation, and the damages were fully evident at the 8-h time point (Figure 3c,d). These images, along with the survival analysis, point to the ability of the compound to induce severe stress resulting in rapid toxicity against the cell lines. This rapid cytotoxicity is intriguing and potentially a useful characteristic for an anticancer scaffold that can be developed against pancreatic cancer. Further studies are required to ascertain the mechanism of action of this compound, and will be reported in due course.

In summary, FUS is a known sesterterpene mycotoxin isolated from the endophytic fungus *F. solani*. This compound showed potent and rapid cytotoxicity against both pancreatic and breast cancer cell lines tested in this study. The complex structure and intriguing biological activity of FUS make it a good target for chemical synthesis and a lead structure for a medicinal chemistry project to develop a new anticancer drug against pancreatic cancer.

## 3. Experimental Section

### 3.1. Collection and Identification of the Plant Material

The aerial part of *A. hookerianum* was collected from Pablakhali, Rangamati, Chittagong Hill tracts, Bangladesh on 10 August 2014 and identified by the taxonomist of Bangladesh National Herbarium, Mirpur, Dhaka. A voucher specimen of the plant has been deposited (Accession no.: DACB 40633) in the herbarium for further reference (Figure S1).

### 3.2. General Experimental Procedures

The NMR spectra were recorded on a Bruker 400 MHz NMR spectrometer using CDCl_3_. The HRMS spectrum was recorded on an Exactive Orbitrap by a Thermo Scientific mass spectrometer at King’s College London, (London, UK), and the data were processed by Thermo XCalibur 2.2. Column chromatography was carried out on silica gel (70–230 mesh and 230–400 mesh, Merck, Darmstadt, Germany). Organic solvents, potato dextrose agar (PDA) medium, and TLC plates were purchased from Merck, Germany.

### 3.3. Isolation of Fungal Material

About 300 g of fresh and healthy parts of the plant (leaves, roots, and petioles) was cut with a sterile scalpel and stored at 4 °C in a sterile polyethene bag prior to use. Endophytic fungi were isolated from the fresh plant parts following the procedure, established at Pharmaceutical Sciences Research Division, BCSIR Laboratories, Dhaka, Bangladesh [11,12,13,14]. Total four endophytic fungi were isolated from different parts of *A. hookerianum* bearing the internal strain no. AHPE-3, AHPE-4 (Figure S2), AHLE-1 and AHLE-4. All the endophytic fungi were taxonomically identified up to genus level on the basis of macroscopic and microscopic morphological characters as *Fusarium* sp. (Figure S3). (AHPE-3), *Fusarium* sp. (AHPE-4), *Colletotrichum* sp. (AHLE-1) and *Colletotrichum* sp. (AHLE-4). The fungus AHPE-4 was selected for further investigation, based on the brine shrimp lethality bioassay data (Figure S4), and was cultured at a large scale to isolate bioactive secondary metabolites.

### 3.4. Molecular Identification of the Endophytic Fungus AHPE-4

For identification and differentiation, the Internal Transcript Spacer regions (ITS4 and ITS5) and the intervening 5.8S rRNA region was amplified and sequenced using electrophoretic sequencing on an ABI 3730 × l DNA analyzer (Applied Biosystems, Waltham, MA, USA) using Big Dye Terminator v 3.1 cycle sequencing kit (Thermo Fisher Scientific, Waltham, MA, USA). The ITS regions of the fungus were amplified using PCR (Hot Start Green Master Mix, Promega, Madison, WI, USA) and the universal ITS primers, ITS4 (5′-TCC GTA GGT GAA CCT GCG G-3′) and ITS5 (5′-GGA AGT AAA AGT CGT AAC AAG G-3′). The PCR products were purified and desalted using the Hot Start Green Master Mix (Cat: M7432, Promega, USA.) and sequenced on an ABI 3730 × l DNA analyzer (Applied Biosystems, USA). The sequences were aligned and prepared with the software Chromas (V 2.6.2, Technelysium, Brisbane, Australia) and matched against the nucleotide-nucleotide database (BLASTn) of the U.S. National Center for Biotechnology Information (NCBI) for final identification of the endophytic isolate. Finally, the sequence data (SI) were deposited in the Gen Bank database (accession number MG75792), which revealed 99% similarity other related fungal isolates of *F. solani* bearing accession numbers KX 497027, KJ863503, AB 190389, AY433805 etc. deposited in NCBI.

### 3.5. Extraction of the Fungal Material and Isolation of FUS

The fungus *F. solani* (AHPE-4), isolated from the petiole of the plant *A. hookerianum*, was cultivated at 28 ± 2 °C for 28 days on potato dextrose agar (PDA). The culture media were extracted with ethyl acetate for seven days in an air-tight, flat-bottom container with occasional shaking and stirring. This procedure was repeated three times to obtain the crude extract. The extract of endophytic fungi was then filtered using sterilized cotton filter followed by Whatman no. 1 filter papers. The solvent was evaporated with a rotary evaporator at low temperature (40 °C–50 °C) and reduced pressure. 

The crude fungal extract (8 gm) was subjected to column chromatography for fractionation on silica gel (70–230 mesh) using gradients of petroleum ether/ethyl acetate, then ethyl acetate, followed by a gradient of ethyl acetate/methanol, and finally methanol, to afford a total of 15 fractions. These fractions were screened by TLC on silica gel under UV light and by spraying with vanillin-H_2_SO_4_ spray reagents. The column fraction of petroleum ether/15% ethyl acetate was subjected to preparative TLC on silica gel (toluene/20% ethyl acetate, 3 developments) to obtain FUS.

#### Fusaproliferin

18 mg, white, amorphous sticky mass; (^1^H-NMR, CDCl_3_): *δ* 2.40 (1H, dd, *J* = 10.8, 13.6 Hz, H-1´), 1.74 (1H, m, H-1″), 5.27 (1H, dd, *J* = 5.0, 10.2 Hz, H-2), 2.30 (1H, m, H-4´), 2.06 (1H, m, H-4″), 2.30 (1H, m, H-5´), 2.11 (1H, m, H-5″), 5.15 (1H, bs, H-6), 2.11 (1H, m, H-8´), 1.82 (1H, d, *J* = 9.2 Hz, H-8″), 1.82 (1H, d, *J* = 9.2 Hz, H-9´), 1.65 (1H, m, H-9″), 4.08 (1H, dd, *J* = 3.4, 9.8 Hz, H-10), 5.40 (1H, bt, H-12), 2.40 (1H, dd, *J* = 10.8, 13.6 Hz, H-13´), 1.95 (1H, m, H-13″), 2.69 (1H, dd, *J* = 2.0, 11.2 Hz, H-14), 2.80 (1H, sextet, H-19), 1.66 (3H, s, H-20), 1.66 (3H, s, H-21), 1.59 (3H, s, H-22), 1.02 (3H, s, H-23), 4.31 (1H, dd, *J* = 8.0, 10.4 Hz, H-24´), 4.27 (1H, dd, *J* = 7.2, 10.4 Hz, H-24″), 1.33 (3H, d, *J* = 6.8 Hz, H-25), 2.06 (3H, s, H-27), 5.56 ( 1H, s, 17- OH). ^13^C-NMR: *δ*_C_ 39.1 (C-1), 121.4 (C-2), 138.2 (C-3), 40.3 (C-4), 23.8 (C-5), 124.3 (C-6), 132.9 (C-7), 34.9 (C-8), 29.7 (C-9), 76.5 (C-10), 136.5 (C-11), 128.9 (C-12), 28.7 (C-13), 49.6 (C-14), 49.0 (C-15), 207.9 (C-16), 147.3 (C-17), 146.7 (C-18), 33.7 (C-19), 15.5 (C-20), 15.3 (C-21), 10.4 (C-22), 16.2 (C-23), 66.4 (C-24), 14.5 (C-25), 170.9 (C-26), 20.9 (C-27). HRESIMS *m/z* 467.2778 [M + Na]^+^ (calcd mass 467.2773, [C_27_H_40_O_5_ + Na]^+^).

### 3.6. Bioassays

#### 3.6.1. Cell Culture 

The MIA PaCa2 (pancreatic adenocarcinoma), BXPC3 (pancreatic adenocarcinoma), MDA-MB-231 (triple-negative breast cancer), MCF-7 (estrogen receptor positive breast cancer) cell lines were obtained from the American Type Culture Collection. The MIA PaCa2 cell line was maintained in Dulbecco’s modified Eagle’s medium (DMEM; Invitrogen, Carlsbad, CA, USA), supplemented with fetal bovine serum (10% *v*/*v*; Invitrogen), horse serum (2.5% *v*/*v*; Invitrogen) and penicillin-streptomycin (1% *v*/*v*, Invitrogen). The BXPC3 cell line was maintained in RPMI-1640 medium (DMEM; Invitrogen), supplemented with fetal bovine serum (10% *v*/*v*; Invitrogen), and penicillin-streptomycin (1% *v*/*v*, Invitrogen). The MDA MB 231 cell line was maintained in Dulbecco’s modified Eagle’s medium (DMEM; Invitrogen), supplemented with fetal bovine serum (10% *v*/*v*; Invitrogen), l-glutamine (2 mM; Invitrogen), non-essential amino acids (1×; Invitrogen) and penicillin-streptomycin (1% *v*/*v*, Invitrogen). The MCF7 cell line was maintained in Eagle’s Minimum Essential medium supplemented with fetal bovine serum (10% *v*/*v*; Invitrogen), 0.01 mg/mL human recombinant insulin and penicillin-streptomycin (1% *v*/*v*, Invitrogen). During seeding, cells were counted using a Neubauer hemocytometer (Assistant, Hanover, Germany) by microscopy (Nikon, Melville, NY, USA) on a non-adherent suspension of cells that were washed in PBS, trypsinized, centrifuged at 8 °C at 8000 rpm for 5 min, and re-suspended in fresh medium.

#### 3.6.2. MTT Assay 

The cells were grown in normal cell culture conditions at 37 °C under a 5% CO_2_ humidified atmosphere using an appropriate medium. The cell count was adjusted to 10^5^ cells/mL and 2500 cells (MDA-MB-231) or 5000 cells (A4 and WI-38) were added per well. The cells were incubated for 24 h, and 1 μL of the appropriate inhibitor concentrations was added to the wells in triplicate. After 96 h of continuous exposure to each compound, the cytotoxicity was determined using the 3-(4,5-dimethylthiazol-2-yl)-2,5-diphenyltetrazolium bromide (MTT) (Lancaster Synthesis Ltd., Morecambe, Lancashire, UK) colorimetric assay. Absorbance was quantified by spectrophotometry at λ = 570 nm (Envision Plate Reader, PerkinElmer, Waltham, MA, USA). IC_50_ values were calculated by a dose-response analysis using the Prism GraphPad Prism^®^ software.

## Figures and Tables

**Table 1:** Cytotoxicity assay results of FUS against human tumor cells (IC_50_ in μM).

Compound/Standard	MIA PaCa 2 (Pancreatic)	BXPC3 (Pancreatic)	MDA MB 231 (Breast)	MCF7 (Breast)	WI 38 (Lung Fibroblast)
FUS	0.13 ± 0.09	0.76 ± 0.24	1.9 ± 0.32	3.9 ± 0.75	18 ± 0.66
Gemcitabine	7.6 ± 0.66	2.2 ± 0.43	NT	NT	NT
Doxorubicin	NT	NT	0.06 ± 0.03	0.02 ± 0.018	NT

## Supplementary Materials

The experimental procedures and ^1^H, ^13^C-NMR, DEPT-135 and HRMS spectra of FUS (PDF) are available online.

## References

[1] Kleeff J., Korc M., Apte M., La Vecchia C., Johnson C.D., Biankin A.V., Neale R.E., Tempero M., Tuveson D.A., Hruban R.H. (2016). Pancreatic cancer. Nat. Rev. Dis. Prim..

[2] Van Cutsem E., Vervenne W.L., Bennouna J., Humblet Y., Gill S., Van Laethem J.-L., Verslype C., Scheithauer W., Shang A., Cosaert J. (2009). Phase III trial of bevacizumab in combination with gemcitabine and erlotinib in patients with metastatic pancreatic cancer. J. Clin. Oncol..

[3] Siriwardena A.K., Siriwardena A.M. (2014). Pancreatic cancer. BMJ Br. Med. J..

[4] Wong M.C.S., Jiang J.Y., Liang M., Fang Y., Yeung M.S., Sung J.J.Y. (2017). Global temporal patterns of pancreatic cancer and association with socioeconomic development. Sci. Rep..

[5] Vincent A., Herman J., Schulick R., Hruban R.H., Goggins M. (2011). Pancreatic cancer. Lancet.

[6] Ouyang L., Luo Y., Tian M., Zhang S.Y., Lu R., Wang J.H., Kasimu R., Li X. (2014). Plant natural products: From traditional compounds to new emerging drugs in cancer therapy. Cell Prolif..

[7] Harvey A.L., Edrada-Ebel R., Quinn R.J. (2015). The re-emergence of natural products for drug discovery in the genomics era. Nat. Rev. Drug Disc..

[8] Kumar A., Patil D., Rajamohanan P.R., Ahmad A. (2013). Isolation, purification and characterization of vinblastine and vincristine from endophytic fungus *Fusarium oxysporum* isolated from *Catharanthus roseus*. PLoS ONE.

[9] Nascimento A.M.d., Conti R., Turatti I.C., Cavalcanti B.C., Costa-Lotufo L.V., Pessoa C., de Moraes M.O., Manfrim V., Toledo J.S., Cruz A.K. (2012). Bioactive extracts and chemical constituents of two endophytic strains of *Fusarium oxysporum*. Rev. Bras. Farmacogn..

[10] Cui Y., Yi D., Bai X., Sun B., Zhao Y., Zhang Y. (2012). Ginkgolide B produced endophytic fungus (*Fusarium oxysporum*) isolated from *Ginkgo biloba*. Fitoterapia.

[11] Khan M.I.H., Sohrab M.H., Rony S.R., Tareq F.S., Hasan C.M., Mazid M.A. (2016). Cytotoxic and antibacterial naphthoquinones from an endophytic fungus, *Cladosporium* sp.. Toxicol. Rep..

[12] Chowdhury N.S., Sohrab M.H., Rony S.R., Sharmin S., Begum M.N., Rana M.S., Hasan C.M. (2016). Identification and bioactive potential of endophytic fungi from *Monochoria hastata* (L.) Solms. Bangladesh J. Bot..

[13] Chowdhury N.S., Sohrab M.H., Rana M.S., Hasan C.M., Jamshidi S., Rahman K.M. (2017). Cytotoxic naphthoquinone and azaanthraquinone derivatives from an endophytic *Fusarium solani*. J. Nat. Prod..

[14] Khan N., Afroz F., Begum M.N., Rony S.R., Sharmin S., Moni F., Hasan C.M., Shaha K., Sohrab M.H. (2018). Endophytic *Fusarium solani*: A rich source of cytotoxic and antimicrobial napthoquinone and aza-anthraquinone derivatives. Toxicol. Rep..

[15] Takemoto K., Kamisuki S., Chia P.T., Kuriyama I., Mizushina Y., Sugawara F. (2014). Bioactive dihydronaphthoquinone derivatives from *Fusarium solani*. J. Nat. Prod..

[16] Shweta S., Zuehlke S., Ramesha B.T., Priti V., Kumar M.P., Ravikanth G., Spiteller M., Vasudeva R., Shaanker U.R. (2010). Endophytic fungal strains of *Fusarium solani*, from *Apodytes dimidiata* E. Mey. ex Arn (Icacinaceae) produce camptothecin, 10-hydroxycamptothecin and 9-methoxycamptothecin. Phytochemistry.

[17] Chakravarthi B., Das P., Surendranath K., Karande A.A., Jayabaskaran C. (2008). Production of paclitaxel by *Fusarium solani* isolated from *Taxus celebica*. J. Biosci..

[18] Kosmidis C., Denning D.W. (2017). Opportunistic and Systemic Fungi. Infectious Diseases.

[19] Randazzo G., Foglianoa V., Ritieni A., Rossin L.M.E., Scarallo A., Segred A.L. (1993). Proliferin, a new sesterterpene from *Fusarium proliferatum*. Tetrahedron.

[20] Ritieni A., Fogliano V., Randazzo G., Scarallo A., Logrieco A., Moretti A., Manndina L., Bottalico A. (1995). Isolation and characterization of fusaproliferin, a new toxic metabolite from Fusarium proliferatum. Nat. Toxins.

[21] Santini A., Ritieni A., Fogliano V., Randazzo G., Mannina L., Logrieco A., Benedetti E. (1996). Structure and absolute stereochemistry of fusaproliferin, a toxic metabolite from Fusarium proliferatum. J. Nat. Prod..

[22] Fotso J., Leslie J.F., Smith S.J. (2002). Production of beauvericin, moniliformin, fusaproliferin, and fumonisins B1, B2, and B3 by fifteen ex-type strains of *Fusarium* Species. Appl. Environ. Microbiol..

[23] Ritieni A., Monti S.M., Moretti A., Logrieco A., Gallo A., Ferracane R., Fogliano V. (1999). Stability of fusaproliferin, a mycotoxin from *Fusarium* spp.. J. Sci. Food Agric..

[24] Logrieco A., Moretti A., Fornelli F., Fogliano V., Ritieni A., Caiaffa M.F., Randazzo G., Bottalico A., Macchia L. (1996). Fusaproliferin production by *Fusarium subglutinans* and its toxicity to *Artemia salina,* SF-9 insect cells, and IARC/LCL 171 human B lymphocytes. Appl. Environ. Microbiol..

[25] Ritieni A., Monti S.M., Randazzo G., Logrieco A., Moretti A., Peluso G., Ferracane R., Fogliano V. (1997). Teratogenic effects of fusaproliferin on chicken embryos. J. Agric. Food Chem..

