# Supplementary material for: Fusaproliferin, a Fungal Mycotoxin, Shows Cytotoxicity against Pancreatic Cancer Cell Lines
Source: Molecules. 2018 Dec 11;23(12):3288. doi: 10.3390/molecules23123288 (PMC6320971; doi:10.3390/molecules23123288)
Supplement: Supplementary file 1 [file molecules-23-03288-s001.pdf]

Fusaproliferin, a Fungal Mycotoxin Shows Cytotoxicity against Pancreatic Cancer Cell Lines.

Nazia Hoque [1,3,4], Choudhury Mahmood Hasan [2], Md. Sohel Rana [3], Amrit Varsha[5], Md. Hossain Sohrab [4*] and Khondaker Miraz Rahman [5*]

[1] Department of Pharmacy, East West University, Dhaka, Bangladesh

[2] Department of Pharmaceutical Chemistry, University of Dhaka, Dhaka-1000, Bangladesh

[3] Department of Pharmacy, Jahangirnagar University, Savar, Dhaka, Bangladesh

[4] Pharmaceutical Sciences Research Division (PSRD), BCSIR Laboratories, Dhaka, Bangladesh

[5] School of Cancer and Pharmaceutical Science, King's College London, 150 Stamford Street, London SE1 9NH, UK

Supporting Information

1. The Plant *Aglaonema hookerianum* Schott

In this study we investigated the fungal endophyte *Fusarium solani,* isolated from petiole of *Aglaonema hookerianum* Schott (Family:Araceae) growing in the forest of Sylhet and Chittagong Hill tracks of Bangladesh.It is a herb, stem erect, 40-50 cm or more tall, 1.5-2.0 cm thick, internodes 1.5-3.0 cm long. Leaves petiolate, petiole up to 24 cm long and grows in the shady areas of deep forest. A large number of tribal populations like Chakma, Marma, Murong, Tongchongya, Tripura, Chak, Khasia, Rheyang, Rakhain, Khumietc live as forest inhabitants in the remote areas throughout Sylhet and the Chittagong Hill Tracts, where there is no or poor modern medical systems of health care. The majority of them are dependent on the traditional system of treatment, which includes various indigenous medicinal plants of those areas [1]. *A. hookerianum,*locally known as Habinishak, is used by the mainstream traditional practitioners of Sylhet district for the treatment of hemorrhoids and arthritis. One tablespoon decoction prepared from the roots of *A. hookerianum* is taken orally twice daily for the treatment of gout. The sap from the root is taken for conjunctivitis and constipation by Chakma community. The leaf extract, applied to the whole

body, is used for the treatment of hysteria by Tanchangya community [2]The petiole of *A. hookerianum* is used in the preparation of ‘Shuktani’, which is an ethno-medico recipe used in the treatment of stomach disorders like diarrhea, indigestion and dysentery by the Sylheti Bengali Community of Barak Valley, Southern Assam, India [3]. Previous phytochemical study revealed the presence of alkaloid, glycoside, tannin, reducing sugar, saponin and gum in the ethanolic extract of *A. hookerianum*. The extract of this plant also showed potent antibacterial and cytotoxic activities [4].

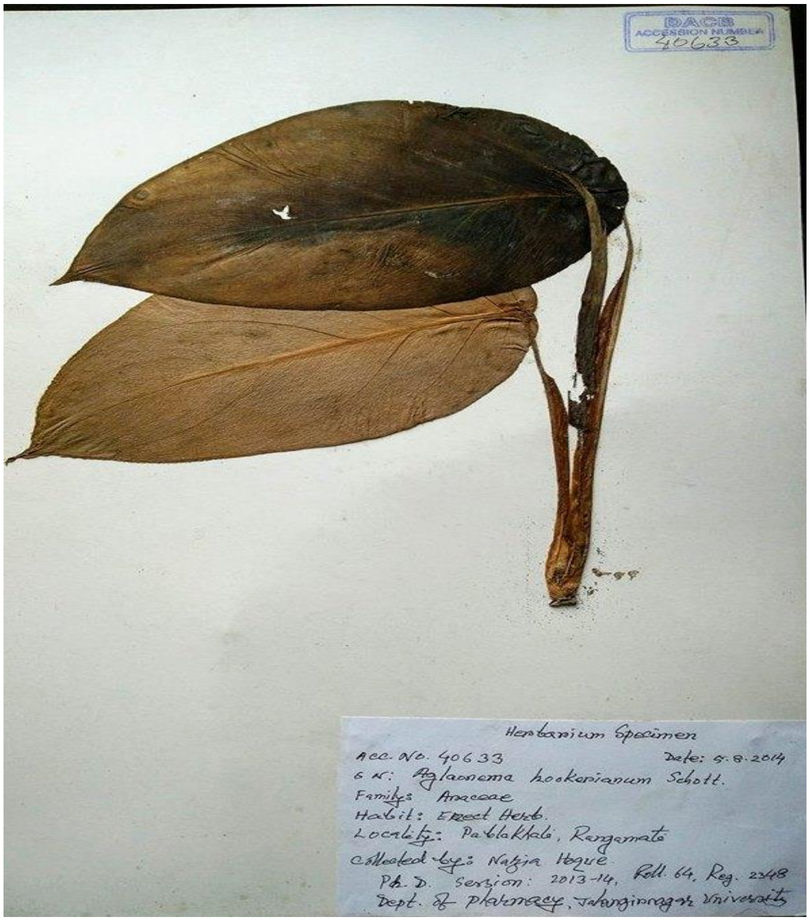

Figure S1. Voucher specimen of the plant *Aglaonema hookerianum* Schott deposited at Bangladesh National Herbarium, Mirpur, Dhaka.**2. Morphological Identification of**

2. Endophytic Fungus AHPE-4

For the identification of endophytic fungal isolates, slides prepared from cultures were stained with lactophenol cotton blue reagent and examined with a bright-field and phase contrast microscope.[6] Identification was based on morphological characteristics such as growth pattern, hyphae, the color of the colony and medium, surface texture, margin character, aerial mycelium, sporulation and production of acervuli, coloration of the medium, and the size and coloration of the conidia using standard identification manuals. The fungi were identified using relevant keys and taxonomic notes from various standard manuals [5].

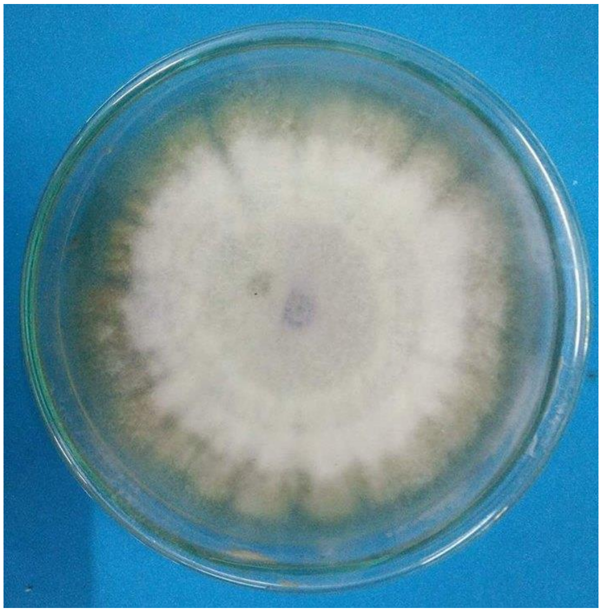

Figure S2. Colony morphology of the endophytic fungus *Fusarium solani* (AHPE-4)

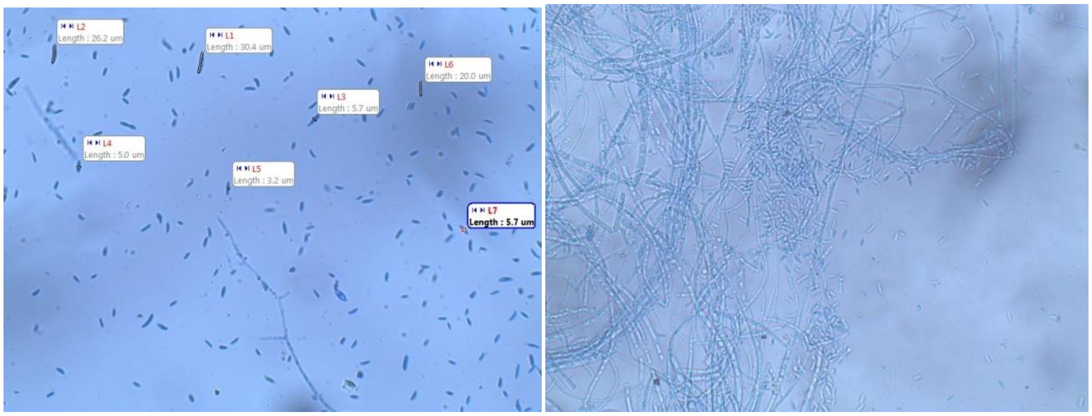

Figure S3. The microscopical view of *Fusarium solani*

3. Molecular Identification of Endophytic Fungus AHPE-4

For identification and differentiation, the Internal Transcript Spacer regions (ITS4 and ITS5) and the intervening 5.8S rRNA region was amplified and sequenced using electrophoretic sequencing on an ABI 3730 x l DNA analyzer (Applied Biosystems, USA) using Big Dye Terminator v 3.1 cycle sequencing kit. **AHPE4_ITS4 seq1 (Organism = *Fusarium solani*)**

CSCGAGGTGTTAGCTACTACGCGATGGAAGCTGCGGCGGGACCGCCACTGTATTTGG
GGGACGGCGCGTGCCCACGGGGGGCTCCSCCGATCCCCMACGCCMGGCCCGGGGG
CCTGAGGGTTGTAATGACSCTCGAACAGGCATGCCCGCCAGAATACTGGCGGGCGC
AATGTGCGTTCAAAGATTCSATGATTCACTGAATTCTGCAATTCACATTACTTATCGC
ATTTCGCTGCGTTCTTCATCGATGCCAGAGCCAAGAGATCCSTTGTTGAAAGTTTTGA
TTTATTTGCTTGTTTTACTCAGAAGATACACTAAAAGAAACAGAGTTAAGGGTCCTC
TGGCGGGGGCGGCCCGTTTTACGGGGCCGTCTATTCCCGCCGAAGCAACGTATAGGT
AAGTTCACAGGGTTGATGAGTTGAATAACTCGGTAATGATCCCTCCGCTGGTTCACC
AACGGAGACCTTGT

Molecular analysis revealed the fungal isolate of AHPE-4 as *Fusarium solani* with the accession number MG75792 deposited in the U.S National Center for Biotechnology Information (NCBI).

4. Preparation of the plant and Initial Fungal Extracts

Total four endophytic fungi were isolated from different parts of *A. hookerianum* named AHPE-3, AHPE-4, AHLE-1 and AHLE-4. The fungus *F. solani*(AHPE-4), isolatedfrom the petiole of the plant *A. hookerianum*Schott after surface sterilization, was cultivated at 28 ± 2 ℃ for 28 days on potato dextrose agar (PDA). The culture media were extracted with ethyl acetate for seven days in an air-tight flat bottom container with occasional shaking and stirring. This procedure was repeated for three times to obtain the crude extract.On theother hand, the powdered plant material (aerial part) of *Aglaonema hookerianum* wasextracted using a dichloromethane: methanol (1:1) solvent system. The crude extracts ofendophytic fungi, as well as the plant, were then filtered using sterilized cotton filter followed by Whatman no. 1 filter papers. The solvent was evaporated with a rotary evaporator at low temperature (40 ℃- 50 ℃) and reduced pressure.

The dried crude extracts were stored at 4 ℃.The extracts of plant as wellas its associated endophytic fungal strains AHPE-3, AHPE-4, AHLE-1 and AHLE-4 were screened for probable cytotoxic activity using brine shrimp lethality bioass

5. Brine Shrimp Lethality Bioassay

Brine shrimp lethality bioassay was used [6] for evaluating cytotoxic activity using different concentrations of each extract using vincristine sulphate as the positive control. The result of the assay is shown in Figure S4. The ethyl acetate extract of *Fusarium solani*(AHPE-4) was found to be most active with an LC_{50} value of 17.05 µg/mL, whereas the positive control vincristine sulphate showed an LC_{50} value of 1.52µg/mL.

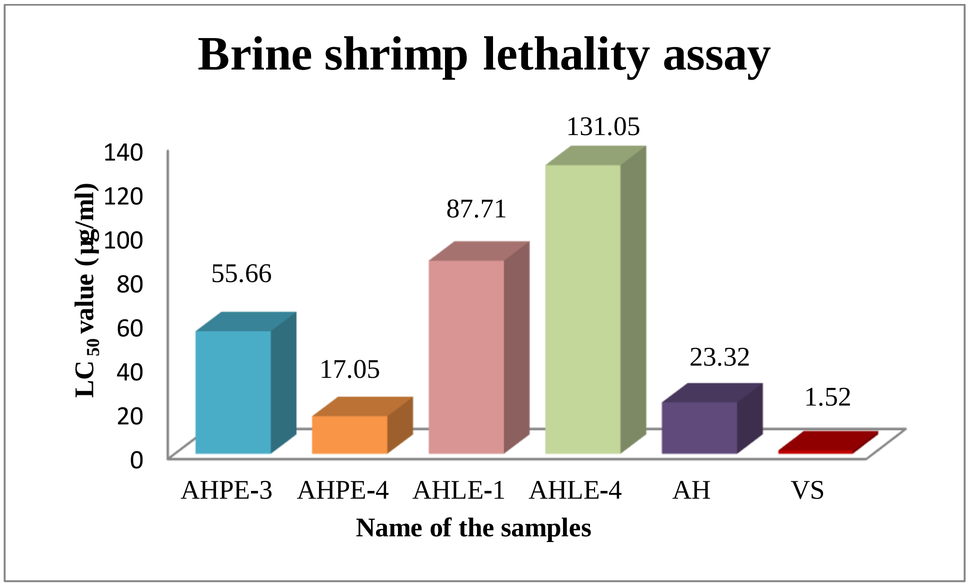

AH- *Aglaonema hookerianum*, VS-Vincristine sulphate

Figure S4. LC_{50} values of the endophytic fungi and the plant *A. hookerianum* in brine shrimp lethality bioassay.

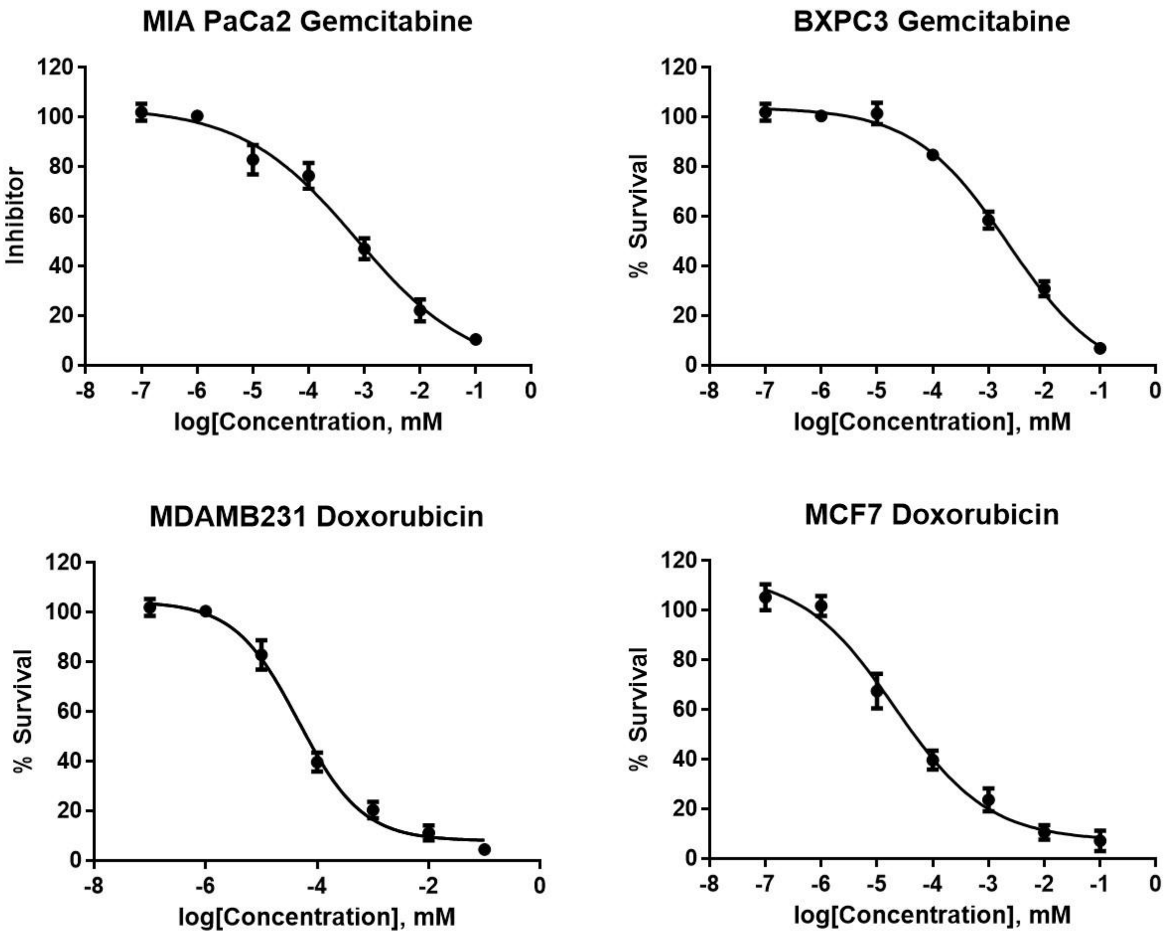

Figure S5. MTT cell-viability assay profile in pancreatic (MIA PaCa2 and BXPC3) treated with gemcitabine and and breast (MDA MB 231 and MCF7) cancer cell lines treated with doxorubicinfor 24 h.

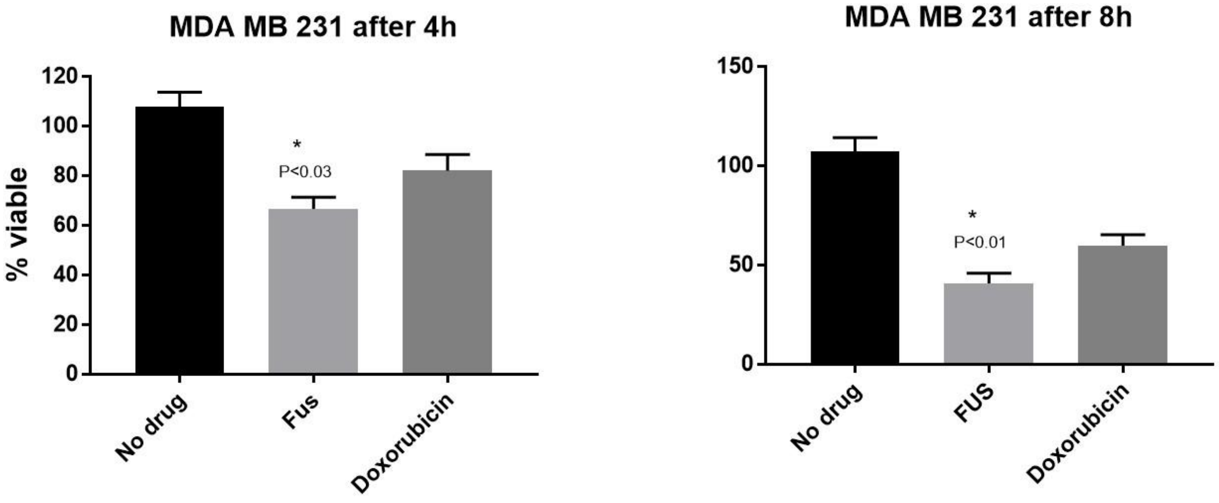

Figure S6. FUS showed statistically significant rapid toxicity against MDA MB 231 cell line after 4h (P<0.03) and 8 h (P<0.01) incubation compared to doxorubicin.

NMR Spectra of Fusaproliferin (FUS)

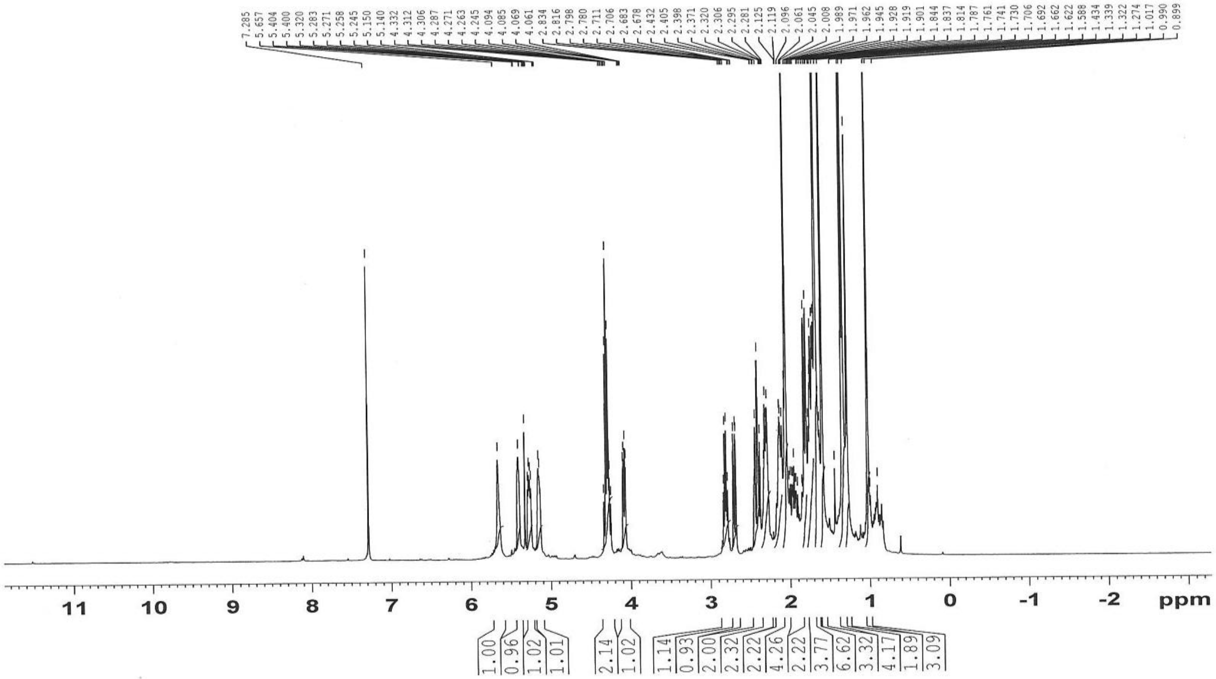

Figure S7. ^{1}H NMR spectrum (400 MHz, $CDCl_3$) of FUS (Full)

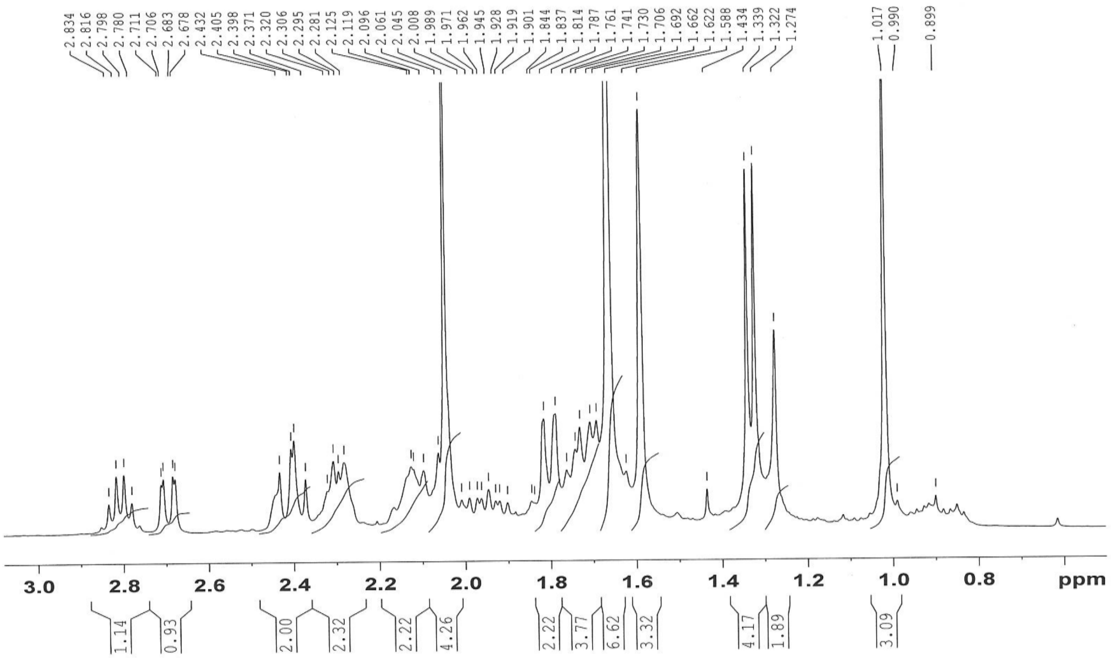

Figure S8. ^{1}H NMR spectrum (400 MHz, $CDCl_3$) of FUS (Expanded)

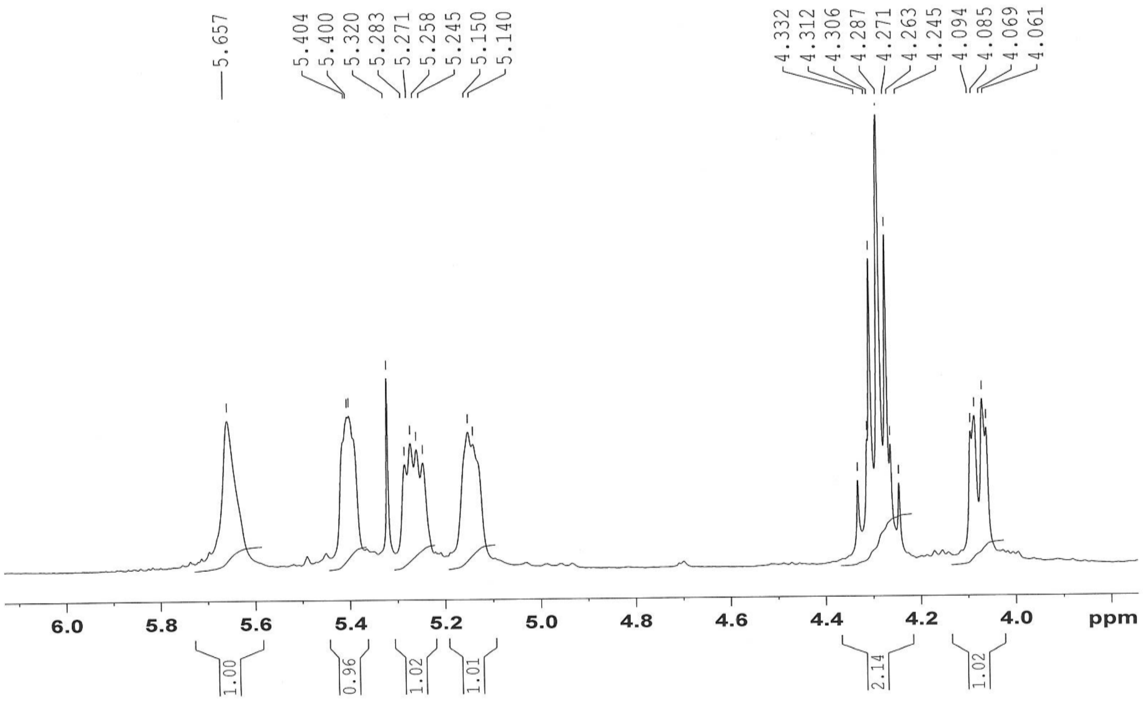

Figure S9. ^{1}H NMR spectrum (400 MHz, $CDCl_3$) of FUS (Expanded)

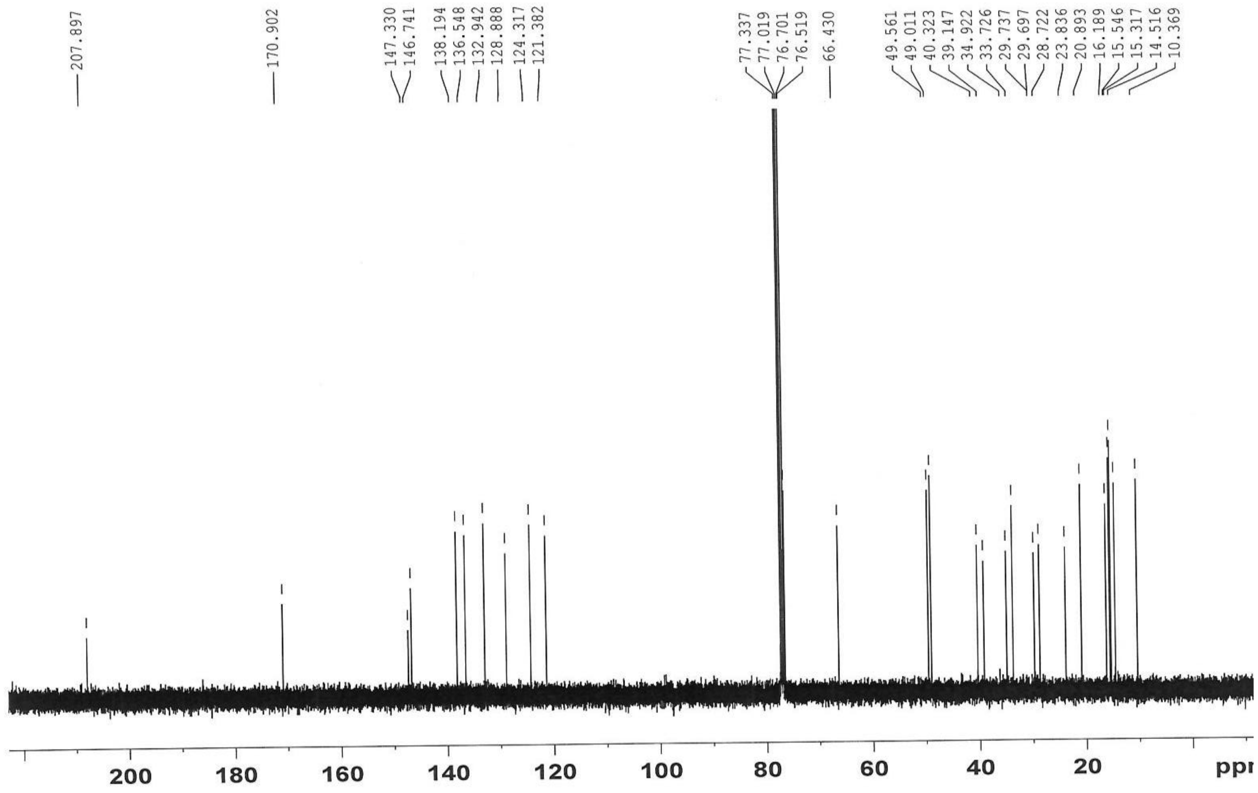

Figure S10. ^{13}C NMR spectrum (100 MHz, $CDCl_3$) of FUS

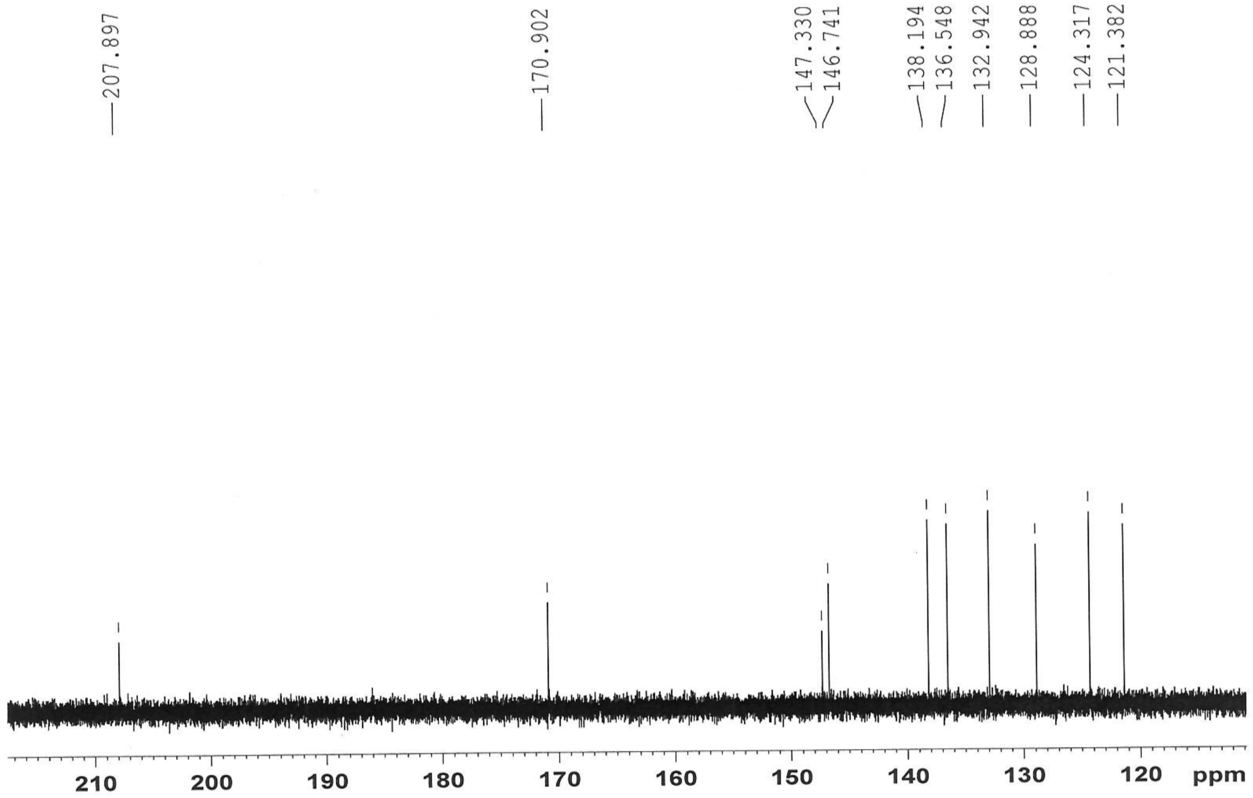

Figure S11. ^{13}C NMR spectrum (100 MHz, CDCL$_3$) of FUS (expanded)

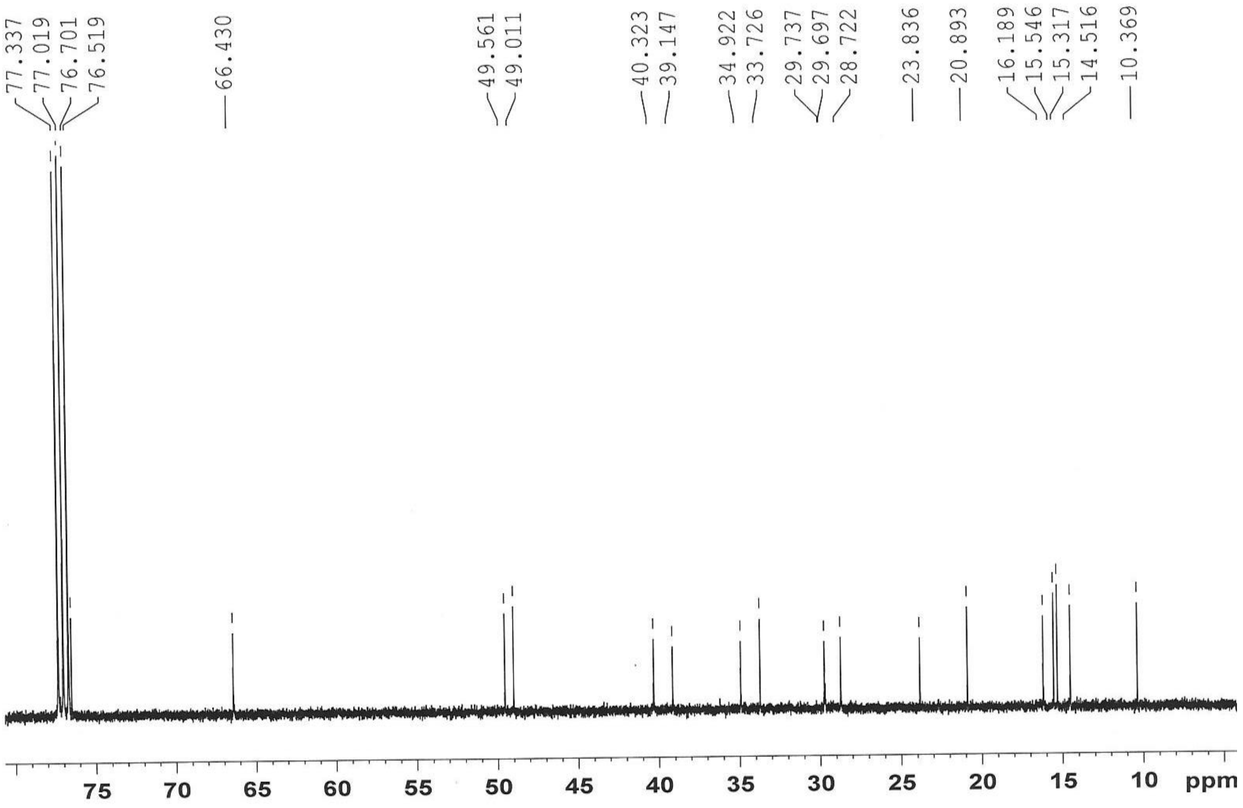

Figure S12. ^{13}C NMR spectrum (100 MHz, $CDCl_3$) of FUS (expanded)

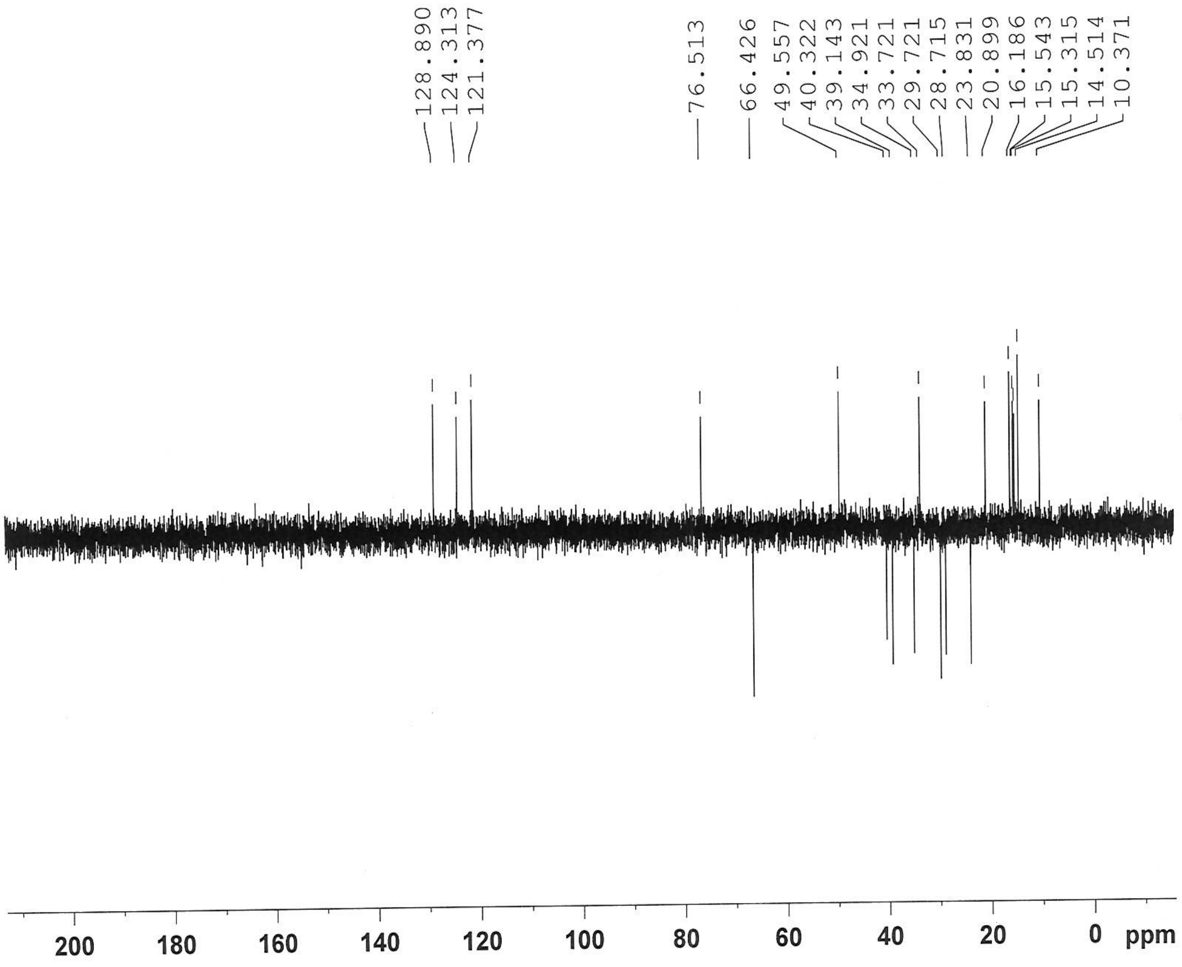

Figure S13. DEPT-135 spectrum (100 MHz, $CDCl_3$) of FUS

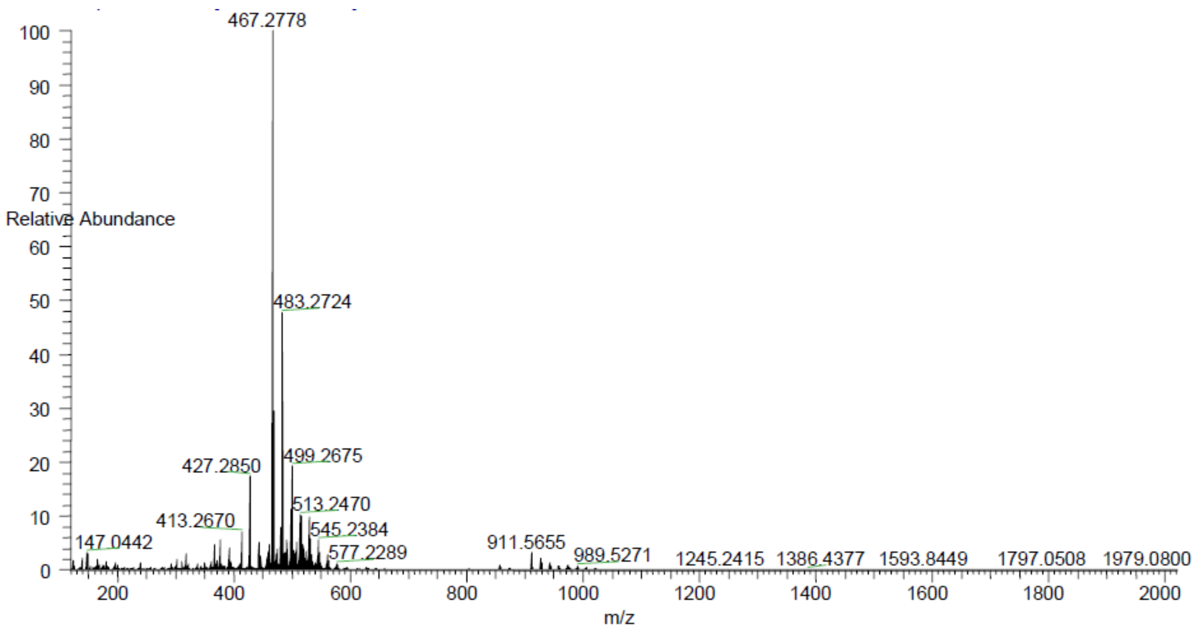

Figure S14. HRMS spectrum of FUS

References

1. Rahman, M. A.; Uddin, S. B.; Wilcock, C. C. Indigenous knowledge of herbal medicine in Bangladesh-Treatment of jaundice by tribal communities of the Hill tract Districts.*Hamdard Medicus* **2003**, *64*, 25-28.
2. Rahmatullah, M.; Rani, S. Pk; Imran, M. A.; Jahan, R., The Khasia tribe of Sylhet District, Bangladesh, and their fast-disappearing knowledge of medicinal plants. *The Journal of Alternative and Complementary Medicine* **2013**, *19,* 599-606.
3. Nath, A.; Maiti, G. G. *Shuktani*- a new ethno-medico recipe among the Sylheti Bengali community of Barak valley, Southern Assam, India. *Indian Journal of Traditional Knowledge* **2012**, *11*, 156-160.
4. Roy, A.; Biswas, S. K.; Chowdhury, A.; Shill, M. C.; Raihan, S. Z.; Muhit, A., Phytochemical screening, cytotoxicity and antibacterial activities of two Bangladeshi medicinal plants. *Pakistan Journal of Biological Sciences* **2011**, *14,* 905-908.
5. Barnett, H. L.; Hunter, B. B. *Illustrated genera of imperfect fungi*.(4th ed). APS Press: St. Paul, Minn. **1998**.
6. Meyer, B.; Ferrigni, N.; Putnam, J.; Jacobsen, L.; Nichols, D.; McLaughlin., Brine shrimp: a convenient general bioassay for active plant constituents. *Planta med.* **1982**, *45,* 31-40.
